# Solid-state NMR characterization of amphomycin effects on peptidoglycan and wall teichoic acid biosyntheses in *Staphylococcus aureus*

**DOI:** 10.1038/srep31757

**Published:** 2016-08-19

**Authors:** Manmilan Singh, James Chang, Lauryn Coffman, Sung Joon Kim

**Affiliations:** 1 Department of Chemistry, Washington University, One Brookings Drive, St. Louis, MO 63130, USA; 2 Department of Chemistry and Biochemistry, Baylor University, One Bear Place #97348, Waco, TX 76798, USA.

## Abstract

Amphomycin and MX-2401 are cyclic lipopeptides exhibiting bactericidal activities against Gram-positive pathogens. Amphomycin and MX-2401 share structural similarities with daptomycin, but unlike daptomycin they do not target bacterial membrane. In this study, we investigate *in vivo* modes of action for amphomycin and MX-2401 in intact whole cells of *Staphylococcus aureus* by measuring the changes of peptidoglycan and wall teichoic acid compositions using solid-state NMR. *S. aureus* were grown in a defined media containing isotope labels [1-^13^C]glycine and L-[ε-^15^N]lysin, L-[1-^13^C]lysine and D-[^15^N]alanine, or D-[1-^13^C]alanine and [^15^N]glycine, to selectively ^13^C-^15^N pair label peptidoglycan bridge-link, stem-link, and cross-link, respectively. ^13^C{^15^N} and ^15^N{^13^C} rotational-echo double resonance NMR measurements determined that cyclic lipopeptide-treated *S. aureus* exhibited thinning of the cell wall, accumulation of Park’s nucleotide, inhibition of glycine utilization for purine biosynthesis, reduction of ester-linked D-Ala in teichoic acids, and reduction of peptidoglycan cross-linking. Whole cell NMR analysis also revealed that *S. aureus*, in presence of amphomycin and MX-2401, maintained the incorporation of D-Ala during peptidoglycan biosynthesis while the incorporation of D-Ala into teichoic acids was inhibited. These effects are consistent with amphomycin’s dual inhibition of both peptidoglycan and wall teichoic acid biosyntheses in *S. aureus*.

Antimicrobial cyclic lipopeptides, including amphomycin^1^, friulimicin^2^, mannopeptimycin^3^, ramoplanin^4^, WAP-8294A_2_^5^, katanosin^6^, plusbacin A_3_^7^,^8^, and daptomycin^9^, exhibit potent antibacterial activities against methicillin-resistant *Staphylococcus aureus* (MRSA), vancomycin-resistant enterococci (VRE), penicillin-gentamicin-erythromycin-resistant *Streptococcus pneumoniae*, and linezolid-quinupristin-dalfopristin-resistant enterococci^10^,^11^,^12^. Amphomycin-class antibiotics, which are a subset of cyclic decapeptides, represent an optimal starting point for the development of novel therapeutic agents against multidrug resistant Gram-positive pathogens. Amphomycin-class antibiotics such as amphomycin, friulimicin, and tshushimycin (Fig. 1a) share the identical decapeptide core structure and differ only by its hydrophobic tail. In amphomycin the hydrophobic tail structure is *anteiso*-tridecenonyl, and in tsushimycin and friulimicin B the tail is *iso*-tetradecenonyl^9^. Optimization of amphomycin through chemical modifications has led to the development of MX-2401 (MIGENIX) with improved activities against VRE, MRSA^13^, and penicillin-resistant *Streptococcus pneumoniae*, where the drug activity remains unaffected by the presence of surfactants that improve pulmonary functions during lung infections^14^.

Amphomycin-class antibiotics and daptomycin share many identical structural features (Fig. 1a). Daptomycin is a cyclic decadepsipeptide approved by FDA in 2003 and it is one of the leading therapeutic agents for treatment of serious skin infections and bacteremia by multidrug resistant pathogens^15^. Both amphomycin and daptomycin share following structural similarities: (1) a 10-amino acid cyclic peptide core structure, (2) a conserved calcium-binding motif with the sequence Asp_4_-X_5-_Asp_6_-Gly_7_, where X_5_ is either D-amino acid or glycine^9^, (3) superimposable D-amino acids (or glycine) at positions 2, 5, 7, and 8 of the cyclic-peptide core, and (4) a hydrophobic tail of similar lengths. One key difference is that daptomycin is a depsipeptide with a lactone linkage instead of a peptide bond between the amino acid positions 1 and 10, (Fig. 1a, yellow highlight).

Despite their structural similarities, daptomycin and amphomycin-class of antibiotics exhibit different modes of action. Daptomycin undergoes a large conformational change upon calcium-ion binding to form a 10 to 16-mer pore structure^16^. The hydrophobic tail of daptomycin is thought to facilitate binding to the membrane by inserting itself into the lipid bilayer^17^ and the ensuing membrane depolarization through K^+^ leakage is thought to be the killing mechanism of daptomycin. In contrast, friulimicin B^2^, amphomycin, and MBX-240^13^ do not depolarize the bacterial membrane even at concentrations ten times the minimum inhibitory concentrations (MIC). Furthermore, cell death induced by amphomycin and MX-2401 preceded the membrane depolarization, suggesting that the membrane depolarization is a consequence of bactericidal activity^13^. Sahl and co-workers, using *in vitro* assays, have shown that friulimicin B^2^ and MX-2401^18^ target the membrane lipid transporter bactoprenol-phosphate (C_55_–P). The structure of amphomycin bound to C_55_–P is unknown, but the x-ray crystal structure of tsushimycin suggests that a binding cleft for the phosphate of C_55_–P may be formed at the dimer interface between the cyclic peptide core structures^19^.

In this study, we investigate *in vivo* mode of action of amphomycin and MX-2401 in intact whole cells of *S. aureus* pair-labeled with [1-^13^C]Gly and L-[ε-^15^N]Lys, L-[1-^13^C]Lys and D-[^15^N]Ala, and D-[1-^13^C]Ala and [^15^N]Gly using solid-state NMR. Cyclic lipopeptides were added to *S. aureus* at mid-exponential growth phase at sub-MIC, then the changes of peptidoglycan (PG) and wall teichoic acid (WTA) compositions were determined by ^13^C{^15^N} and ^15^N{^13^C} rotational-echo double resonance (REDOR) NMR^20^.

## Results

### MX-2401 does not induce ATP leakage in *S. aureus*

**Figure 1:**
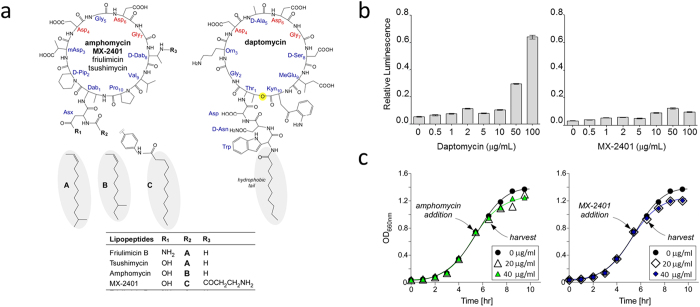
Daptomycin and amphomycin have different modes of action. (**a**) Chemical structures of cyclic decapeptide antimicrobial agents: friulimicin, amphomycin, tsushimycin, MX-2401, and daptomycin. All of these cyclic lipopeptides share a ten-membered peptide core structure with hydrophobic tail. The hydrophobic tail group in amphomycin is *anteiso*-tridecenonyl, and in tsushimycin and friulimicin B it is *iso*-tetradecenonyl. All cyclic decapeptides exhibit calcium dependent bactericidal activity against Gram-positive bacteria and have a conserved calcium-binding motif sequence Asp_4_-Asp_6_-Gly_7_. Daptomycin is a cyclic-depsipeptide with a lacton linkage highlighted in yellow. (**b**) ATP-leakage assay performed on overnight culture of *S. aureus*. Daptomycin induces leaking of ATP from *S. aureus*, but only at a high drug concentration (100 μg/mL). MX-2401 did not cause the leakage in *S. aureus* at any concentrations. Thus, MX-2401 does not disrupt the membrane despite sharing structural similarities with daptomycin. (**c**) Growth curves of *S. aureus* treated with amphomycin (left), and MX-2401 (right) as monitored by the optical density (OD) at 660 nm. Cyclic lipopeptides were added to final concentrations of 20 or 40 μg/mL at mid-exponential growth (OD_660nm_ 0.7). Addition of cyclic lipopeptides at these concentrations did not significantly perturb the growth. The cells were harvested for NMR analysis after one doubling time (60 minutes) of incubation with drugs at the approximate OD_660nm_ of 0.9.

ATP-leakage assay was performed on overnight culture of *S. aureus* (Fig. 1b) by adding daptomycin or MX-2401 to final drug concentrations of 0, 0.5, 1, 2, 5, 10, 50, and 100 μg/mL (Fig. 1b). Daptomycin induced ATP leakage was observed at higher concentrations (50 and 100 μg/mL), but MX-2401 did not show significant ATP leakage at any concentration. Solid-state NMR samples were prepared from harvested *S. aureus* at mid-exponential growth phase after treated with amphomycin or MX-2401 to final drug concentrations of 20 or 40 μg/mL. *S. aureus* grown in the presence of amphomycin and MX-2401 at both drug concentrations did not exhibit any observable growth perturbation (Fig. 1c) nor ATP leakage (Fig. 1b).

### Amphomycin-treated *S. aureus* exhibit cell wall thinning and Park’s nucleotide accumulation

**Figure 2:**
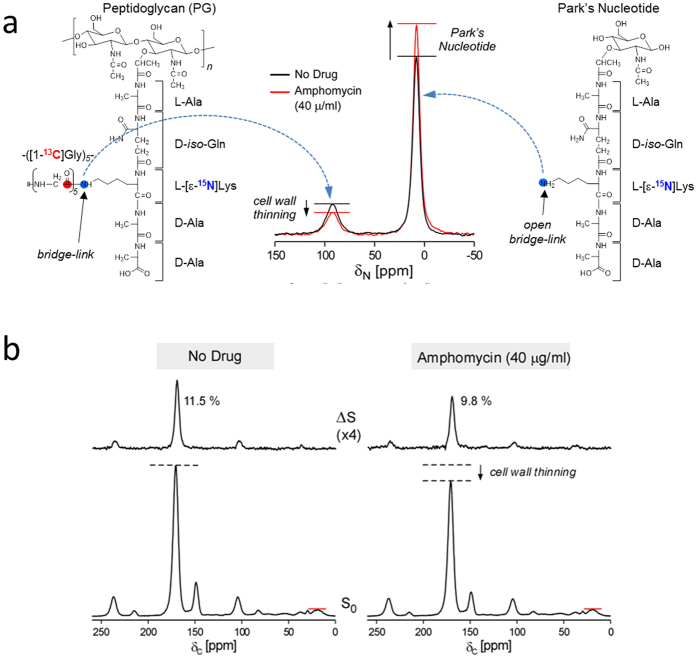
Amphomycin-treated *S. aureus* exhibit cell wall thinning and Park’s nucleotide accumulation. (**a**) Incorporation of [1-^13^C]Gly and L-[ε-^15^N]Lys to peptidoglycan (PG). ^15^N-CPMAS NMR spectra from amphomycin-treated whole cells of *S. aureus* (40 μg/mL, red line) and untreated whole cells (black line). Amphomycin-treated *S. aureus* show reduced lysyl-ε-amide peak at 95 ppm due to cell-wall thinning, and increased lysyl-ε-amine peak at 10 ppm due to accumulation of Park’s nucleotide. These effects are consistent with amphomycin inhibition of cell wall biosynthesis by targeting steps at or prior to PG transglycosylation. Each spectrum is the result of 20,480 accumulated scans. (**b**) ^13^C{^15^N} REDOR spectra from whole cells of *S. aureus* at 1.6 ms of dipolar evolution. S_0_ spectra are at the bottom, and ΔS spectra at the top. Spectra are normalized to natural abundance peaks from 10 to 30 ppm range. The 170-ppm glycyl peak intensities in S_0_ are directly proportional to the amount of [1-^13^C]Gly in *S. aureus*. The decrease in 170-ppm peak intensity in the S_0_ spectrum of amphomycin (40 μg/mL) treated *S. aureus* is consistent with thinning of the cell wall. Amphomycin-treated *S. aureus* also show decrease in the 149-ppm peak intensity in the S_0_ indicating reduction of [1-^13^C]Gly into purine biosynthesis. In ΔS spectra (top) only covalently bonded ^13^Cs from ^13^C-^15^N spin pairs unique to PG bridge-links are detected at 1.6 ms dipolar evolution. 170-ppm dephasing (ΔS/S_0_) decreases from 11.5% to 8.9% with addition of amphomycin, which indicates the reduction in the proportion of L-[ε-^15^N]Lys due to incorporation of unlabeled L-Lys into PG stem structure. Each spectrum is the result of 10,240 accumulated scans.

Growing *S. aureus* in defined media containing [1-^13^C]Gly and L-[ε-^15^N]Lys enabled quantification of the covalent ^13^C-^15^N pair labeling exclusively formed at the PG bridge-links (Fig. 2a). ^15^N-CPMAS NMR spectra of amphomycin treated (Fig. 2a, red) and untreated (Fig. 2a black line) *S. aureus* whole cells for the drug concentration of 40 μg/ml are overlaid for comparison. Incorporation of L-[ε-^15^N]Lys into PG with a pentaglycine bridge attached results in a lysyl-ε-amide unique to PG with the chemical shift of 95 ppm. Incorporation of L-[ε-^15^N]Lys into Park’s nucleotide (cytoplasmic PG precursor) as lysyl-ε-amine can be observed as a peak with the chemical shift of 10 ppm due to its open bridge-link. Amphomycin-treated *S. aureus* show the reduction of lysyl-ε-amide at 95 ppm and increase in lysyl-ε-amine at 10 ppm. The reduction of 95 ppm corresponds to the loss of PG through cell wall thinning, and the increased lysyl-ε-amine at 10 ppm corresponds to the accumulation of Park’s nucleotide.

^13^C{^15^N} REDOR spectra of whole cells of *S. aureus* with and without amphomycin treatment after 1.6 ms of dipolar evolution are shown in Fig. 2b. *S*_*0*_ spectra (bottom) are normalized to natural abundance peaks from 10 to 30 ppm range. The 170-ppm glycyl peak intensities in *S*_*0*_ spectra (bottom) are directly proportional to the total amount of [1-^13^C]Gly found in *S. aureus*. The reduced glycyl-carbonyl carbon in the *S*_*0*_ of amphomycin-treated *S. aureus* indicates the thinning of cell wall due to inhibition of PG biosynthesis. Covalently bonded ^13^C-^15^N spin pairs unique to the PG bridge-link are selected in Δ*S* spectra after 1.6 ms dipolar evolution. Reduced Δ*S* 170-ppm intensity in amphomycin-treated *S. aureus* is the result of increased incorporation of endogenous unlabeled L-Lys into PG-stem structure from biosynthesis of cytoplasmic PG precursors. The 149-ppm peak in the *S*_*0*_ is due to the metabolism of [1-^13^C]Gly into purine biosynthesis. The reduction of 149-ppm peak in amphomycin-treated *S. aureus* indicates decreased amount of [1-^13^C]Gly entering purine biosynthesis pathway due to the heavy demand on [1-^13^C]Gly by PG biosynthesis pathway.

### Amphomycin-treated *S. aureus* show reduced PG cross-linking

**Figure 3:**
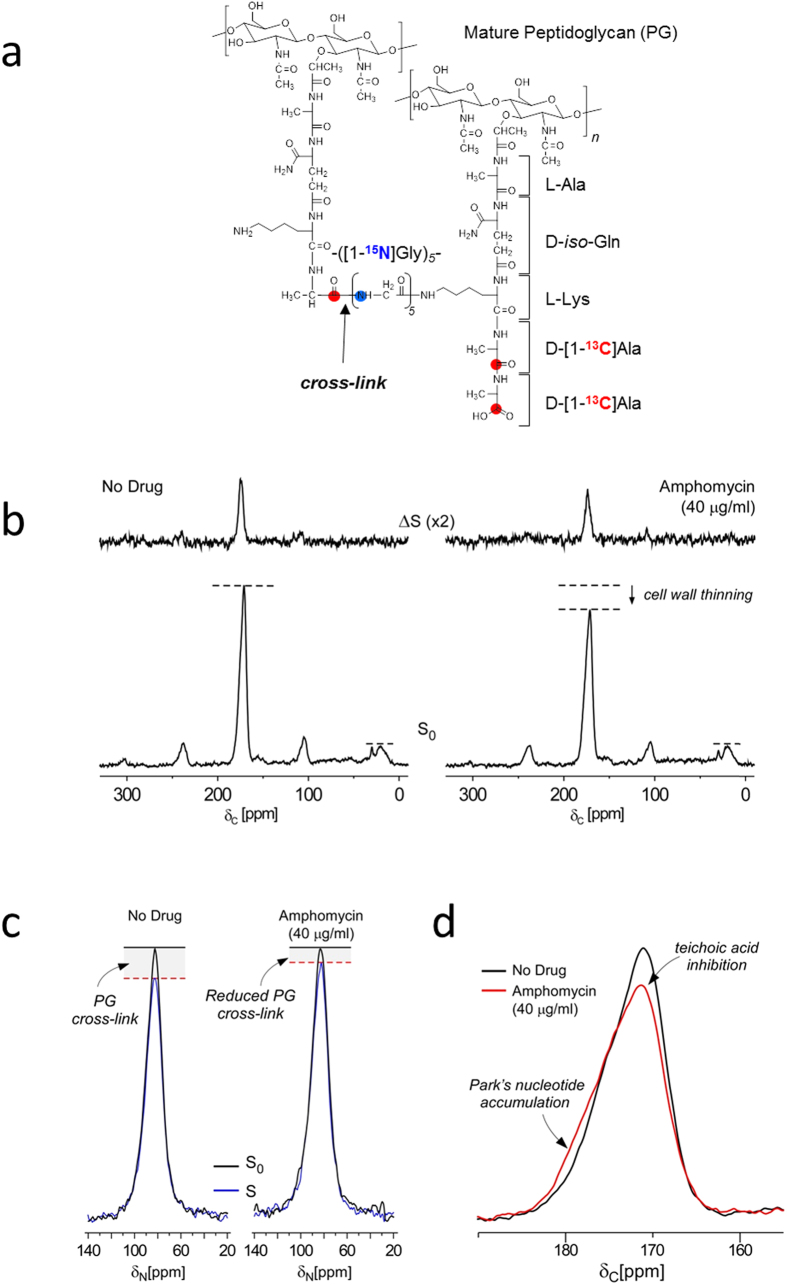
Amphomycin-treated *S. aureus* exhibit reduced peptidoglycan crosslinking. (**a**) Incorporation of D-[1-^13^C]Ala (red dots) and [^15^N]Gly (blue dot) and the 13C-^15^N pair labeled in the PG cross-link. (**b**) ^15^N{^13^C} REDOR spectra after dipolar evolution of 1.6 ms from *S. aureus* labeled with D-[1-^13^C]Ala and [^15^N]Gly. Full-echo spectra (*S*_*0*_) are shown in black and the REDOR dephased spectra (*S*) are in red, while spectra from the sample with amphomycin (40 μg/mL) are on the right and without on the left. (**c**) The difference in peak intensities (*S*_*0*_-*S*) measures the relative number of cross-links per PG pentaglycyl bridging segment. Amphomycin reduced cross-linking, which is consistent with mild inhibition of transpeptidation. (**d**) Enlarged carbonyl-carbon regions of the ^13^C{^15^N} REDOR full-echo spectra of amphomycin treated (red) and untreated (black) *S. aureus*. The lineshape of spectra from amphomycin treated cells shows increased 178 ppm contribution from the carboxyl-carbonyl carbon of D-[1-^13^C]Ala from Park’s nucleotide unincorporated into PG by cross-linking. The decreased 172 ppm contribution is due to the reduced amount of ester-linked D-[1-^13^C]Ala in WTA.

*S. aureus* was grown in defined media with D,L-[1-^13^C]Ala and [^15^N]Gly to selectively label ^13^C-^15^N pair of the PG cross-link in order to determine the effect of amphomycin on transpeptidation step of PG biosynthesis (Fig. 3a). ^13^C{^15^N} REDOR spectra of whole cells of *S. aureus* grown in presence (40 μg/mL) and absence of amphomycin with dipolar evolution of 1.6 ms are shown in Fig. 3b. The S_0_ spectra are normalized to natural abundance peaks from 10 to 30 ppm range. The S_0_ spectrum of amphomycin-treated *S. aureus* shows reduction of D,L-[1-^13^C]Ala incorporation into PG. In Δ*S* spectra, 175-ppm peak intensity is proportional to the total amount of covalently bonded ^15^N-^13^C spin pairs found in *S. aureus*. Although the intensity of Δ*S* 175-ppm peak includes contributions from [^15^N]Gly-L-[1-^13^C]Ala sequences found in proteins, the primary contribution to the intensity comes from cross-linked PG. Alanine racemase inhibitor alaphosphin has been used to ensure selective labeling of PG with D-[1-^13^C]Ala and to remove protein contribution to the ΔS 175-ppm intensity^8^,^21^,^22^,^23^,^24^,^25^,^26^,^27^,^28^; however, alaphosphin was not used to eliminate potential synergic effect of alaphosphin with amphomycin. Amphomycin-treated *S. aureus* showed reduction in ΔS 175-ppm peak intensity, which is directly proportional to the total number of PG cross-links. This reduced PG cross-linking in amphomycin-treated *S. aureus* is apparent in the enlarged carbonyl carbon region of the S_0_ overlaid with the dephased (S) spectra (Fig. 3c), where the difference between 175-ppm intensities of S_0_ and S is directly proportional to the total number of PG cross-links.

### Amphomycin and MX-2401 treated *S. aureus* exhibit reduced ester-linked D-Ala

The lineshape of S_0_ 175 ppm alanyl-carbonyl carbon peak of amphomycin-treated *S. aureus* (Fig. 3d, red line) is different from untreated sample (Fig. 3d, black line). Overlaid spectra show that the 175-ppm peak from amphomycin-treated *S. aureus* has a broadened shoulder due to the increase in 178-ppm contribution from the penultimate D-Ala carbonyl carbon of accumulated Park’s nucleotide^8^. In contrast, diminished 170-ppm shoulder of amphomycin-treated *S. aureus* spectrum indicates reduction in the incorporation of ester-linked D-Ala incorporation into lipoteichoic acid (LTA) and WTA.

**Figure 4:**
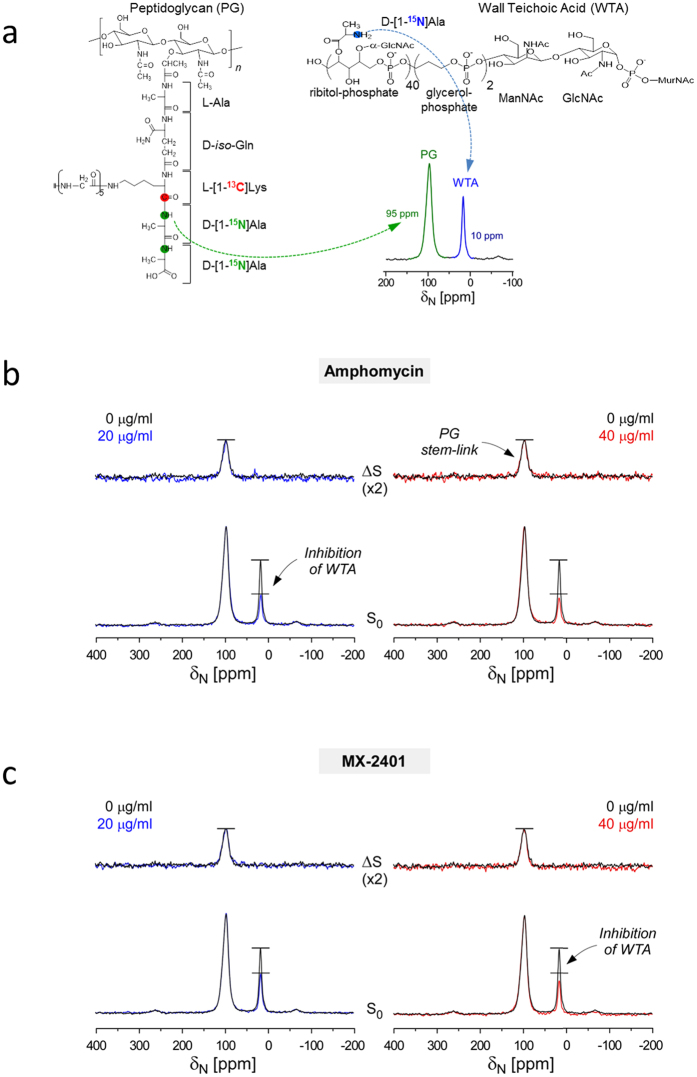
Amphomycin and MX-2401 inhibit D-Ala incorporation into wall teichoic acid. (**a**) Incorporation of D-[^15^N]Ala into PG and wall teichoic acid (WTA) cell wall monitored using ^15^N-CPMAS NMR. D-[^15^N]Ala in PG has an alanyl amide at 95 ppm in the ^15^N-CPMAS spectrum, while in WTA ester-linked D-[^15^N]Ala is an amine at 10 ppm (lower right). The effect of cyclic lipopeptides on WTA biosynthesis is monitored by measuring the ratio between PG to WTA. (**b**) ^15^N{^13^C} REDOR spectra of *S. aureus* treated with amphomycin (0, 20, and 40 μg/mL) at 1.6 ms dipolar evolution. (**c**) ^15^N{^13^C} REDOR spectra of whole cells of *S. aureus* treated with MX-2401 (0, 20, and 40 μg/mL) at 1.6 ms dipolar evolution. The full-echo spectrum (S_0_) is at the bottom and the REDOR difference (ΔS) at the top. *S*_*0*_ spectra are normalized to equal alanyl-amide peak at 95 ppm of ΔS spectra. Addition of amphomycin and MX-2401 resulted in the decrease in alanyl-amine peak at 10 ppm in S_0_ spectra, indicating reduction in the amount of ester-linked D-Ala in teichoic acid.

To determine the degree of amphomycin inhibiting the incorporation of ester-linked D-Ala into teichoic acids, *S. aureus* were grown in defined media containing L-[1-^13^C]Lys and D-[^15^N]Ala (Fig. 4a). The growth was carried out in the presence of alaphosphin (5 μg/mL) to prevent the racemic scrambling of D-[^15^N]Ala into L-[^15^N]Ala^24^. Incorporation of D-[^15^N]Ala into PG was visible as an amide peak at 95 ppm in the ^15^N-CPMAS spectrum, and D-[^15^N]Ala incorporation into WTA as an amine peak at 10 ppm (Fig. 4a). ^15^N{^13^C} REDOR spectra at 1.6 ms of dipolar evolution for *S. aureus* treated with amphomycin (Fig. 4b) and MX-240 (Fig. 4c) are shown. In ΔS spectra, covalently bonded carbons from ^15^N-^13^C spin pairs (L-[1-^13^C]Lys-D-[^15^N]Ala) from PG stem-links are observed at 95 ppm. The ΔS 95-ppm peak intensity is directly proportional to the total number of PG stem-links in *S. aureus*. The absence of change in ΔS 95-ppm intensity for *S. aureus* grown in the presence of antibiotics indicated that amphomycin and MX-2401 did not inhibit the incorporation of D-[^15^N]Ala into PG-stem structure. In contrast, a large reduction of 10-ppm peak intensity in S_0_ spectra of amphomycin and MX-2401 treated *S. aureus* which corresponds to ester-linked D-[^15^N]Ala found in WTA and LTA was observed. Thus, amphomycin and MX-2401 at sub-MIC concentrations significantly impair the incorporation of ester-linked D-[^15^N]Ala into teichoic acids in *S. aureus*.

## Discussion

We investigated *in vivo* mode of action of amphomycin and MX-2401 by measuring the changes in cell wall composition of intact whole-cells of *S. aureus* using solid-state NMR. It was found that amphomycin-treated *S. aureus* exhibit cell wall thinning (Figs 1b, 2b and 3b) concomitant with the accumulation of Park’s nucleotide (Figs 1a and 3d), which indicates that amphomycin inhibits cell wall biosynthesis by targeting steps at or prior to PG transglycosylation. These observations are consistent with the proposed membrane transporter targeting mode of action for amphomycin^1^,^29^. Recent *in vitro* studies using bacterial membrane extracts have shown that amphomycin^18^ and friulimicin B^2^ form Ca^2+^ dependent complexes with bactoprenol-phosphate (C_55_-P) to inhibit the formation of lipids I, II, and III. Lipids I and II are the membrane transporters for PG biosynthesis, and lipid III is for WTA biosynthesis.

As C_55_-P is a central lipid transporter shared by PG and WTA biosyntheses, we investigated the comparative effects of amphomycin and MX-2401 on PG and WTA biosyntheses. Accurate biochemical quantification of PG and WTA are difficult, especially for WTA analysis^30^ which must rely on a series of purification steps dependent on the release of WTA from the cell wall. In contrast, solid-state NMR approach provides direct and accurate measurement of D-[^15^N]Ala incorporation into PG and WTA in intact whole cells. We were able to clearly quantify the amount of D-[^15^N]Ala incorporated into PG by measuring the amount of PG stem-links using ^13^C{^15^N} REDOR (Fig. 3), and D-[^15^N]Ala incorporation into ester-linked D-Ala in WTA by ^15^N{^13^C} REDOR NMR (Fig. 4). The NMR analysis of whole cells yielded a surprising result that *S. aureus* differently utilize D-Ala for PG and WTA biosyntheses under antibiotic stress. ^15^N{^13^C} REDOR NMR spectra (Fig. 4b,c) show in amphomycin and MX-2401 treated bacteria, the amount of ester-linked D-[^15^N]Ala is reduced by 50% when compared to untreated *S. aureus*. As *S. aureus* were harvested following 60 minutes of growth, one doubling time, in presence of antibiotic, 50% reduction in ester-linked D-[^15^N]Ala represents total inhibition of D-[^15^N]Ala incorporation into teichoic acids by amphomycin and MX-2401. The reduction in ester-linked D-[^15^N]Ala can be due to either i) direct inhibition of WTA biosynthesis, and/or ii) inhibition of D-Ala incorporation by targeting of D-alanylation pathway specific to WTA biosynthesis. Although solid-state NMR measurements cannot differentiate between these two mechanisms, it is clear that amphomycin and MX-2401 had a direct and consequential effect on wall teichoic acid composition. Surprisingly, amphomycin and MX-2401 had no visible effect on the incorporation of D-[^15^N]Ala into PG stem-link (Fig. 4b,c), which suggests at the cost of maintaining PG biosynthesis in amphomycin and MX-2401 treated *S. aureus* is decreased WTA biosynthesis.

Whole cell analysis by solid-state NMR indicates that *in vivo* mode of action for amphomycin is complex. For example, glycine metabolism towards purine biosynthesis is suppressed in amphomycin treated *S. aureus* (Fig. 2b) presumably due to routing of all available glycine to PG biosynthesis pathway in response to the cell wall inhibition. While the downstream effect of purine biosynthesis inhibition by amphomycin is unknown, presumably it would directly alter the overall metabolism of bacteria. Another interesting effect of amphomycin on whole cell is the reduction of PG cross-linking (Fig. 3c). This reduction was unexpected as the targeting of C_55_-P by amphomycin is unlikely to have any direct inhibitory effects on transpeptidase activity. We propose two possible explanations for the amphomycin inhibition of PG cross-linking, although there may be multiple ways these scenarios could manifest themselves. First, amphomycin’s inhibitory effect on PG cross-linking is an indirect effect from inhibition of ester-linked D-Ala incorporating into WTA (and potentially inhibiting WTA biosynthesis), which can result in mislocalization of penicillin-binding protein 4 (PBP4). PBP4 is the transpeptidase responsible for high levels of PG cross-linking observed in *S. aureus*. WTA has been shown to be essential in the recruitment of PBP4 to the septum during the cell division. In WTA deficient *tagO*-deletion mutant of *S. aureus*, PBP4 is shown to be dispersed over the cell surface resulting in reduced PG cross-linking^31^. Second, amphomycin can directly interfere with transpeptidase activity through steric interference by directly binding to PG and forming a complex. Antibiotics that target PG with possessing a hydrophobic sidechain such as oritavancin^32^ and plusbacin A_3_^8^ reduce PG cross-linking in *S. aureus*. Similar mechanism of action can be envisioned for amphomycin, but the binding of amphomycin to PG remains yet to be demonstrated.

## Methods

### Growth and labeling of whole cells

Starter culture of *S. aureus* (ATCC 6538P) grown overnight in 5 ml of trypticase soy broth in 37 °C at 250 rpm was added (1% final volume) to two one-liter flasks, each containing 250 mL of *S. aureus* Standard Medium (SASM) supplemented with calcium chloride to final concentration of 50 μg/mL^28^. The detailed protocol for the defined medium SASM has been described previously^33^,^34^,^35^. Bridge-links and cross-links of PG are selectively ^13^C-^15^N isotope pair labeled (Fig. 2) by addition of [1-^13^C]Gly and L-[ϵ-^15^N]Lys, or [^15^N]Gly and D-[1-^13^C]Ala in presence of alanine racemase inhibitor alaphosphin (5 mg/L) to SASM. WTA was ^15^N-isotope labeled (Fig. 3, right) by replacing the natural isotopic abundance L-Ala and L-Lys of SASM with isotope-labeled D,L -[^15^N]Ala and L- [1-^13^C]Lys.

At mid-exponential growth of *S. aureus* with optical density at 660 nm (OD_660_) of 0.7, amphomycin or MX-2401 was added to final drug concentrations of 20 and 40 μg/mL. Cells were harvested after 60 minutes of further growth (OD_660_ of 0.9) by centrifugation at 8,000 g for 10 min at 4 °C in Sorvall GS-3 rotor. The cells were washed twice with 50 mL of ice-cold 40 mM triethanolamine buffer, pH 7.0, and resuspended in 10 mL of water and lyophilized.

### Dipolar Recoupling

Cell wall compositions were analyzed by ^13^C{^15^N} and ^15^N{^13^C} REDOR NMR. REDOR is the solid-state NMR method that recouples heteronuclear dipolar interactions under magic-angle spinning^20^, which is used to determine dipolar couplings dependent on inter-nuclear distances. REDOR is a difference experiment in which two spectra are collected, one in the absence of heteronuclear dipolar coupling (full echo, S_0_ spectrum), and the other in the presence of the coupling (dephased echo, S spectrum). In the S_0_ spectrum, dipolar dephasing is refocused over a single rotor period due to the spatial averaging by the motion of rotor under magic-angle spinning. In the *S* spectrum, spin part of the dipolar interaction is manipulated by the application of rotor-synchronized dephasing π-pulses that prevent full refocusing. Dipolar evolution over a rotor period in the S spectrum results in reduced peak intensity for dipolar coupled spin pairs. The difference in signal intensity (REDOR difference, ΔS = S_0_ − S) for the observed spin (^13^C or ^15^N) in two parts of the REDOR experiment is directly related to the heteronuclear dipolar coupling from which the corresponding distance to the dephasing spin is determined. The difference spectra are typically collected as a function of N rotor periods to map out the dipolar evolution.

### Solid-state NMR Spectrometer

REDOR experiments were performed on whole cells at 7.0 T (300 MHz for ^1^H, 75 MHz for ^13^C, and 30 MHz for ^15^N) provided by 89-mm bore Oxford (Cambridge, U.K.) superconducting solenoids. The four-frequency transmission-line probe used in the 7.0-T spectrometer had a 14-mm long, 9-mm inner-diameter sample coil, while that used in the 4.7-T spectrometer had a 17-mm long, 8.6-mm inner-diameter sample coil. Both probes were equipped with Chemagnetics/Varian magic-angle spinning ceramic stator, and samples were spun at room temperature at 5 kHz (maintained within ±2 Hz). Radio-frequency pulses were produced by 1-kW Kalmus, ENI, and American Microwave Technology power amplifiers, each under active control; π-pulse lengths were 10 μs for both ^13^C and ^15^N. Proton-carbon and proton-nitrogen matched cross-polarization transfers were at 50 kHz for 2 ms. Proton dipolar decoupling during signal acquisition was 105 kHz. Standard XY-8 phase cycling^37^ was used for all refocusing and dephasing pulses. The re-cycle delay period was 2 seconds during which each amplifier produced a 300-μsec test pulse. Resulting diode-detected voltages were compared to the reference voltage previously calibrated. Differences were used to correct the drives of power amplifiers for the next repetition of the REDOR pulse sequence. Combination of active control of the amplifiers^38^ and alternate-scan data acquisition for each pair of REDOR spectra (S and S_0_) eliminated long-term drifts in the performance of the spectrometer. The normalized REDOR difference (ΔS/S_0_) is a direct measure of dipolar coupling, and this quantity was calculated using modified Bessel function expressions given by Mueller *et al*.^38^ and de la Caillerie and Fretigny^39^ for an IS spin-^1^/_2_ pair.

### ATP-leakage assay

ATP-leakage assay was performed on overnight cultures of *S. aureus* grown in TSB harvested at OD_600_ 1.5. Cells were first pelleted, then resuspended in phosphate buffered saline (PBS) supplemented with 20 mM Ca^2+^. Cyclic lipopeptide was added to the suspension to final drug concentrations of 0, 0.5, 1, 2, 5, 10, 50, and 100 μg/mL and the cells incubated for 20 min at 37 °C. After the incubation, bacteria were pelleted and removed from the suspension by centrifuging. The amount of leaked ATP in the supernatant was quantified by directly adding 100 μL of CellTiter-Glo® 2.0 reagent (Promega, Madison WI) to equal volume of supernatant. After 10 minutes of equilibration, luminescence given off by the mixture was measured using Fluoroskan Ascent FL Luminometer (Thermo Scientific) with the integration time of 200 ms.

## Additional Information

**How to cite this article**: Singh, M. *et al*. Solid-state NMR characterization of amphomycin effects on peptidoglycan and wall teichoic acid biosyntheses in *Staphylococcus aureus*. *Sci. Rep*. **6**, 31757; doi: 10.1038/srep31757 (2016).

## References

[b1] Tanaka H. . Studies on bacterial cell wall inhibitors. II. Inhibition of peptidoglycan synthesis *in vivo* and *in vitro* by amphomycin. Biochim Biophys Acta 497, 633–640 (1977). 407940 10.1016/0304-4165(77)90283-5

[b2] Schneider T. . The lipopeptide antibiotic Friulimicin B inhibits cell wall biosynthesis through complex formation with bactoprenol phosphate. Antimicrob. Agents Chemother. 53, 1610–1618 (2009). 19164139 10.1128/AAC.01040-08 PMC2663061

[b3] Ruzin A. . Mechanism of action of the mannopeptimycins, a novel class of glycopeptide antibiotics active against vancomycin-resistant gram-positive bacteria. Antimicrob Agents Chemother 48, 728–738 (2004). 14982757 10.1128/AAC.48.3.728-738.2004 PMC353120

[b4] Hu Y., Helm J. S., Chen L., Ye X. Y. & Walker S. Ramoplanin inhibits bacterial transglycosylases by binding as a dimer to lipid II. J Am Chem Soc 125, 8736–8737 (2003). 12862463 10.1021/ja035217i

[b5] Kato A. . A new anti-MRSA antibiotic complex, WAP-8294A. I. Taxonomy, isolation and biological activities. J Antibiot (Tokyo) 51, 929–935 (1998). 9917006 10.7164/antibiotics.51.929

[b6] Maki H., Miura K. & Yamano Y. Katanosin B and plusbacin A_3_, inhibitors of peptidoglycan synthesis in methicillin-resistant *Staphylococcus aureus*. Antimicrob. Agents Chemother. 45, 1823–1827 (2001). 11353632 10.1128/AAC.45.6.1823-1827.2001 PMC90552

[b7] Shoji J. . Isolation and characterization of new peptide antibiotics, plusbacins A1-A4 and B1-B4. J. Antibiot. 45, 817–823 (1992). 1500345 10.7164/antibiotics.45.817

[b8] Kim S. J. . The isotridecanyl side chain of plusbacin-A_3_ is essential for the transglycosylase inhibition of peptidoglycan biosynthesis. Biochemistry 52, 1973–1979 (2013). 23421534 10.1021/bi4000222 PMC3628776

[b9] Baltz R. H., Miao V. & Wrigley S. K. Natural products to drugs: daptomycin and related lipopeptide antibiotics. Nat. Prod. Rep. 22, 717–741 (2005). 16311632 10.1039/b416648p

[b10] Weis F., Beiras-Fernandez A. & Schelling G. Daptomycin, a lipopeptide antibiotic in clinical practice. Curr. Opin. Investig. Drugs 9, 879–884 (2008). 18666036

[b11] Schrag S. J. . Emergence of *Streptococcus pneumoniae* with very-high-level resistance to penicillin. Antimicrob. Agents Chemother. 48, 3016–3023 (2004). 15273115 10.1128/AAC.48.8.3016-3023.2004 PMC478489

[b12] Baysallar M., Kilic A., Aydogan H., Cilli F. & Doganci L. Linezolid and quinupristin/dalfopristin resistance in vancomycin-resistant enterococci and methicillin-resistant *Staphylococcus aureus* prior to clinical use in Turkey. Int. J. Antimicrob. Agents 23, 510–512 (2004). 15120733 10.1016/j.ijantimicag.2003.09.029

[b13] Dugourd D. . Antimicrobial properties of MX-2401, an expanded-spectrum lipopeptide active in the presence of lung surfactant. Antimicrob. Agents Chemother. 55, 3720–3728 (2011). 21576435 10.1128/AAC.00322-11 PMC3147646

[b14] Craig W. A., Andes D. R. & Stamstad T. *In vivo* pharmacodynamics of new lipopeptide MX-2401. Antimicrob. Agents Chemother. 54, 5092–5098 (2010). 20855736 10.1128/AAC.00238-10 PMC2981244

[b15] Kern W. V. Daptomycin: first in a new class of antibiotics for complicated skin and soft-tissue infections. Int. J. Clin. Pract. 60, 370–378 (2006). 16494659 10.1111/j.1368-5031.2005.00885.x

[b16] Scott W. R., Baek S. B., Jung D., Hancock R. E. & Straus S. K. NMR structural studies of the antibiotic lipopeptide daptomycin in DHPC micelles. Biochim. Biophys Acta. 1768, 3116–3126 (2007). 17945184 10.1016/j.bbamem.2007.08.034

[b17] Lakey J. H. & Ptak M. Fluorescence indicates a calcium-dependent interaction between the lipopeptide antibiotic LY146032 and phospholipid membranes. Biochemistry 27, 4639–4645 (1988). 2844233 10.1021/bi00413a009

[b18] Rubinchik E. . Mechanism of action and limited cross-resistance of new lipopeptide MX-2401. Antimicrob. Agents Chemother. 55, 2743–2754 (2011). 21464247 10.1128/AAC.00170-11 PMC3101398

[b19] Bunkoczi G., Vertesy L. & Sheldrick G. M. Structure of the lipopeptide antibiotic tsushimycin. Acta Crystallogr. D Biol. Crystallogr. 61, 1160–1164 (2005). 16041082 10.1107/S0907444905017270

[b20] Weldeghiorghis T. K. & Schaefer J. Compensating for pulse imperfections in REDOR. J. Magn. Reson. 165, 230–236 (2003). 14643704 10.1016/j.jmr.2003.08.005

[b21] Kim S. J., Singh M., Sharif S. & Schaefer J. Cross-link formation and peptidoglycan lattice assembly in the FemA mutant of *Staphylococcus aureus*. Biochemistry 53, 1420–1427 (2014). 24517508 10.1021/bi4016742 PMC3985804

[b22] Sharif S., Kim S. J., Labischinski H., Chen J. & Schaefer J. Uniformity of glycyl bridge lengths in the mature cell walls of Fem mutants of methicillin-resistant *Staphylococcus aureus*. J. Bacteriol. 195, 1421–1427 (2013). 23335411 10.1128/JB.01471-12 PMC3624537

[b23] Kim S. J., Tanaka K. S., Dietrich E., Rafai Far A. & Schaefer J. Locations of the hydrophobic side chains of lipoglycopeptides bound to the peptidoglycan of *Staphylococcus aureus*. Biochemistry 52, 3405–3414 (2013). 23607653 10.1021/bi400054p PMC3778154

[b24] Kim S. J., Singh M., Preobrazhenskaya M. & Schaefer J. *Staphylococcus aureus* peptidoglycan stem packing by rotational-echo double resonance NMR spectroscopy. Biochemistry 52, 3651–3659 (2013). 23617832 10.1021/bi4005039 PMC3796188

[b25] Sharif S., Singh M., Kim S. J. & Schaefer J. *Staphylococcus aureus* peptidoglycan tertiary structure from carbon-13 spin diffusion. J. Am. Chem. Soc. 131, 7023–7030 (2009). 19419167 10.1021/ja808971c PMC2778264

[b26] Sharif S., Kim S. J., Labischinski H. & Schaefer J. Characterization of peptidoglycan in fem-deletion mutants of methicillin-resistant *Staphylococcus aureus* by solid-state NMR. Biochemistry 48, 3100–3108 (2009). 19309106 10.1021/bi801750u PMC2785074

[b27] Kim S. J., Singh M. & Schaefer J. Oritavancin binds to isolated protoplast membranes but not intact protoplasts of *Staphylococcus aureus*. J. Mol. Biol. 391, 414–425 (2009). 19538971 10.1016/j.jmb.2009.06.033 PMC2747642

[b28] Kim S. J., Matsuoka S., Patti G. J. & Schaefer J. Vancomycin derivative with damaged D-Ala-D-Ala binding cleft binds to cross-linked peptidoglycan in the cell wall of *Staphylococcus aureus*. Biochemistry 47, 3822–3831 (2008). 18302341 10.1021/bi702232a PMC2778263

[b29] Tanaka H., Oiwa R., Matsukura S. & Omura S. Amphomycin inhibits phospho-N-acetylmuramyl-pentapeptide translocase in peptidoglycan synthesis of Bacillus. Biochem Biophys Res Commun. 86, 902–908 (1979). 106855 10.1016/0006-291x(79)91797-2

[b30] Xia G. . Glycosylation of wall teichoic acid in *Staphylococcus aureus* by TarM. J. Biol. Chem. 285, 13405–13415 (2010). 20185825 10.1074/jbc.M109.096172 PMC2859500

[b31] Atilano M. L. . Teichoic acids are temporal and spatial regulators of peptidoglycan cross-linking in Staphylococcus aureus. Proc Natl Acad Sci USA 107, 18991–18996 (2010). 20944066 10.1073/pnas.1004304107 PMC2973906

[b32] Kim S. J. . Oritavancin exhibits dual mode of action to inhibit cell-wall biosynthesis in *Staphylococcus aureus*. J. Mol. Biol. 377, 281–293 (2008). 18258256 10.1016/j.jmb.2008.01.031 PMC2276640

[b33] Kim S. J. . Rotational-echo double resonance characterization of vancomycin binding sites in *Staphylococcus aureus*. Biochemistry 41, 6967–6977 (2002). 12033929 10.1021/bi0121407

[b34] Tong G. . Structure and dynamics of pentaglycyl bridges in the cell walls of *Staphylococcus aureus* by ^13^C-^15^N REDOR NMR. Biochemistry 36, 9859–9866 (1997). 9245418 10.1021/bi970495d

[b35] Kim S. J., Cegelski L., Preobrazhenskaya M. & Schaefer J. Structures of *Staphylococcus aureus* cell-wall complexes with vancomycin, eremomycin, and chloroeremomycin derivatives by ^13^C{^19^F} and ^15^N{^19^F} rotational-echo double resonance. Biochemistry 45, 5235–5250 (2006). 16618112 10.1021/bi052660s PMC2504515

[b36] Gullion T., Baker D. B. & Conradi M. S. New, compensated Carr-Purcell sequences. J. Magn. Reson. 89, 479–484 (1990).

[b37] Stueber D., Mehta A. K., Chen Z., Wooley K. L. & Schaefer J. Local order in polycarbonate glasses by ^13^C{^19^F} Rotational-Echo Double-Resonance NMR. J. Polym. Sci., Part B: Polym. Phys. 44, 2760–2775 (2006).

[b38] Mueller K. T., Jarvie T. P., Aurentz D. J. & Roberts B. W. The REDOR transform: direct calculation of internuclear couplings from dipolar-dephasing NMR data. Chem. Phys. Lett. 242, 535–542 (1995).

[b39] de la Caillerie J.-B. d. E. & Fretigny C. Analysis of the REDOR signal and inversion. J. Magn. Reson. 133, 273–280 (1998). 9716468 10.1006/jmre.1998.1462

